# Congresses: what do you crave?

**DOI:** 10.1590/2177-6709.22.5.009-010.edt

**Published:** 2017

**Authors:** David Normando

**Affiliations:** 1 Adjunct professor, Universidade Federal do Pará (UFPA), School of Dentistry, Belém, Pará, Brazil. Coordinator, Universidade Federal do Pará (UFPA), Graduate program in Dentistry, and ABO-Pará, Specialization course in Orthodontics, Belém, Pará, Brazil.

*“The pleasure of banquets does not lie in the abundance of food, but, rather, in the company of friends and in conversation.”* Cicero, Roman orator

The word *congress* (from the Latin *congressus*) means a meeting of people with the purpose of discussing a topic of interest to all. 

Every October in odd-numbered years, the Brazilian Association of Orthodontics and Facial Orthopedics (ABOR) holds its Congress. Its purpose is to bring together researchers, professors, specialists and graduate students for whom Orthodontics is the Holy Grail. This year, the 11^th^ ABOR International Congress will be held in Belém do Pará, Brazil - for the first time, in the Brazilian Amazon. 

In those lands, we find the *Museu do Encontro* (“Museum of the Encounter”), located in the *Forte do Presépio* (“Fort of the Holy Manger”). In this museum, we find a print by Hans Staden that depicts a gathering of Tupinamba indigenous people for one more celebration. With their origin in the Tupi ethnic groups, those indigenous people lived along almost all Brazilian coastal line before the arrival of the Portuguese. The Tupinamba have been known for their fighting spirit and their gatherings, which, at first, seemed to be cannibalistic rituals. This anthropophagic practice, common to many Tupinamba indigenous peoples, was followed for reasons that went beyond the biological function of feeding oneself. The consumption of human flesh was the result of symbolic actions that unfolded in situations of war between different tribes. Some of these peoples believed that the power of the captured fighter could be incorporated by means of this ritual. 

Congresses are events that have been held since the advent of science. The basic objective of scientific congresses is to publicize technical and scientific knowledge at a faster pace than through scientific publications, which follow a longer and slower path. Experts from all over the world gather to present, in a direct and richly illustrated manner, their views on certain topics. Some of these presentations are based on science; others, not so much - refuting or questioning scientific evidences. 

When the systems of sound/image transmission emerged in the academic world, in the beginning of the 1990s, even before the advent of the Internet for the same purpose, Professor Omar Gabriel da Silva Filho was asked whether technology would bring about the demise of scientific gatherings. With his characteristic wisdom, he answered that nothing would replace face-to-face contacts between people; that the chance to talk with a lecturer and colleagues directly in a meeting or congress is an activity that will last as long as our species exists. Omar has always believed that there is a considerable amount of feelings in a scientific event. To feel the intonation and emotion in the voice, to enjoy the possibility of approaching lecturers - some, true idols -, to be in contact with ideas and products even before they are launched, to present data about our own studies still under development and, principally, to reunite with friends that time does not take away, all makes us believe in Omar’s lucid prediction. 

Congresses are, therefore, meetings that keep us socialized and intertwined through friendship. Science is, unquestionably, our food; however, in the same way as our Tupinamba ancestors, and as described in a popular Brazilian song, *“we don’t crave food only; we crave food, entertainment and art”*. 

How about you? What do you crave? 

David Normando - editor-in-chief (davidnormando@hotmail.com)
